# In Search of Critically Endangered Species: The Current Situation of Two Tiny Salamander Species in the Neotropical Mountains of Mexico

**DOI:** 10.1371/journal.pone.0034023

**Published:** 2012-04-02

**Authors:** Adriana Sandoval-Comte, Eduardo Pineda, José L. Aguilar-López

**Affiliations:** Red de Biología y Conservación de Vertebrados, Instituto de Ecología, A. C., Xalapa, Veracruz, México; University of Massachusetts, United States of America

## Abstract

Worldwide, one in every three species of amphibian is endangered, 39 species have gone extinct in the last 500 years and another 130 species are suspected to have gone extinct in recent decades. Of the amphibians, salamanders have the highest portion of their species in one of the risk categories, even higher than the frogs. To date there have been few studies that have used recent field data to examine the status of populations of endangered salamanders. In this study we evaluate the current situation of two tiny salamanders, *Parvimolge townsendi* and *Thorius pennatulus*, both of which are distributed at intermediate elevations in the mountains of the northern Neotropics and are considered to be critically endangered; the first has been proposed as possibly extinct. By carrying out exhaustive surveys in both historical and potentially suitable sites for these two species, we evaluated their abundance and the characteristics of their habitats, and we estimated their potential geographic distribution. We visited 22 sites, investing 672 person-hours of sampling effort in the surveys, and found 201 *P. townsendi* salamanders in 11 sites and only 13 *T. pennatulus* salamanders in 5 sites. Both species were preferentially found in cloud forest fragments that were well conserved or only moderately transformed, and some of the salamanders were found in shade coffee plantations. The potential distribution area of both species is markedly fragmented and we estimate that it has decreased by more than 48%. The results of this study highlight the importance of carrying out exhaustive, systematic field surveys to obtain accurate information about the current situation of critically endangered species, and help us better understand the crisis that amphibians are facing worldwide.

## Introduction

In recent years amphibians have been receiving particular attention on the conservation scene because of the crisis they are facing worldwide. One in three of the amphibian species on the planet is endangered, 37 species are thought to have gone extinct since 1500 C.E., two more species are now extinct in the wild [1] and a further 130 species are thought to have gone extinct in recent decades [2]. Caudate amphibians (salamanders) have the highest percentage of species that are at some degree of risk (50%)—even greater than that of frogs and toads (32%)—however the current plight of salamanders and the causes of their decline have received comparatively little attention [3]–[5].

Lungless salamanders (Plethodontidae) make up 68% of the caudate amphibians in the world [6] and it is in the Neotropics, particularly in the mountainous regions of Central America and central-southern Mexico, where this family has undergone its greatest diversification [7], [8]. Of the many Plethodontidae salamanders that inhabit the region and face conservation problems, *Thorius pennatulus* and *Parvimolge townsendi*, the first described by Cope [9] and the latter by Dunn [10], are two tiny salamander species that exemplify the situation many other amphibians are facing. Both species are considered critically endangered (CR) by the International Union for the Conservation of Nature [1], and are classified as threatened (*P. townsendi*) and under special protection (*T. pennatulus*) on the official Mexican list [11]. The distribution of these species is restricted (<5000 km^2^) and they both inhabit the mountainous region of central Veracruz in Mexico, at intermediate elevations between 800 and 2,000 m a.s.l. in cloud forest and tropical semideciduous forest [5], [12], [13]. Historically both species were considered abundant, but a few years ago a warning was sounded about the dramatic declines in their populations with the main cause cited as the destruction and modification of their natural habitat [3], [5], [14]–[16]. It was recently discovered that forty years ago, the fungus *Batrachochytrium dendrobatis* was found in the region, including infected *T. pennatulus* [17], suggesting that this chytrid fungus might be another important agent in the decline in the amphibians of the region. After several unsuccessful search expeditions in different years [3], [5], *P. townsendi* was classified as possibly extinct by the IUCN [15]. A single *T. pennatulus* was observed in the late 1990s, one in 2004 and another in 2006 [14]. In spite of the difficult situation these two species are in, some studies mention finding one or two *P. townsendi* specimens [18]–[20], and the observation of *T. pennatulus* [21], mostly in localities where they had not been found previously.

To study species that in recent years or in the last few decades have been difficult to observe or even impossible to find, such as those classified as in critical danger of going extinct and particularly those considered as possibly extinct, it is necessary to carry out exhaustive field surveys to verify their status [22], [23]. While the search should focus on historical localities [22], [24], it is also necessary to search in sites that are as yet unexplored and have a habitat similar to the localities of prior records for the species, as these are the sites that can potentially maintain populations of the species.

From August to December 2010, several specialists from around the world launched a search for 100 species of amphibians that have not been seen in a decade or longer and may now be extinct. The search resulted in the rediscovery of only four of the 100 amphibians sought, one of them was the salamander *Chiropterotriton mosaueri* in Mexico [25]. Other rediscoveries of two plethodontid salamanders, in Guatemala, have also been reported recently [26].

Given the worrisome scenario for both of these species of salamanders and their sporadic appearance after several years of not having been seen, in this study we decided to carry out extensive, exhaustive field searches for them to corroborate their presence (or document their absence) in localities where they have been reported historically and in new sites with habitat that is potentially suitable for them and, based on these observations, provide information about their abundance, the characteristics of their habitat and distribution; all in order to provide solid, current bases to further the conservation of both species.

## Materials and Methods

### Ethics statement

To conduct this work we first obtained permits from the Mexican wildlife agency (permit number: SGPA/DGVS/03665/06). Manipulation of animals in the field was minimal in all cases. We measured each encountered individual using an electronic caliper, weighed with a pesola balance (5 g) and photographed with a Fujifilm digital camera, and the microhabitat where it was found was noted. After we recorded these different traits, individuals were returned to the same site where they were found.

### Historical biological data

The first stage of this study involved compiling historical biological data for the two species, paying particular attention to the number of specimens recorded, the date and the collection location. We consulted the *Sistema Nacional de Información sobre Biodiversidad* (SNIB) database, curated by CONABIO, that of the Global Biodiversity Information Facility (GBIF) as well as HerpNet's database (see list of information sources in Table S1), and the specialized literature [18]–[21]. After creating a single database from this information, all of the records were checked to eliminate any inconsistencies or duplicates, to correct the geographic coordinates of imprecise localities and georeference localities for which the coordinates were not given. The final database for *P. townsendi* lists 219 specimens, and of these 193 specified the collection date and 217 specified where they had been collected (26 sites with unique coordinates, see Table S2). The final database for *T. pennatulus* lists 985 specimens, and of these 679 specified the collection date and 821 specified where they had been collected (10 sites, see Table S2). For the latter species, there was insufficient data for 164 of the records so they were not used in this study.

### Searching for salamanders in the field

The survey sites for the species of salamander studied were selected based on two criteria. They were either 1) historical sites (i.e. sites where these species had been recorded in the past, or sites no more than 1.5 km from sites where they had been recorded in the past) or 2) they were new sites where the habitat was like the original forest or where it was similar to sites where the species had been recorded. To identify new sites that were potentially suitable habitats for populations of these species of salamander, we looked for bioclimate attributes similar to those of the historical localities, and vegetation types similar to cloud forest, tropical semideciduous forest or shade coffee plantations. To identify potential sites, we modeled habitat suitability using the MaxEnt program, version 3.3.3a [27]. The program uses a machine-learning algorithm and the technique of maximum entropy to determine the optimal probability distribution based on a set of environmental constraints [27]. The program uses two input resources: the localities of the species record (presence-only data) and digital layers of the environmental conditions of a given region. The localities that we used were the historical collection localities for each species (see Table S2) and the environmental data layers were the 19 climate layers from the WorldClim project [28] at a resolution of 0.0083° (∼1 km^2^). The predictive models for both species were generated using the automatic mode and all of the presence points to train the model and none to test it (because the small number of occurrence records for both species). The logistic format was used to obtain the values of habitat suitability (continuous probability from 0 to 1). The resulting values were transformed to binary presence-absence values, with a threshold value which was the minimum probability in which all the records of species presence would be included (minimum training presence). For *P. townsendi* the threshold value was 0.347, and for *T. pennatulus* it was 0.424. Once we had estimated the area with the bioclimate characteristics appropriate for each species, we checked current aerial images of the region to find sites with forest within the predicted area and later, during site visits, we corroborated the presence of the forest and identified the vegetation type. In the end, 22 sites located in the central region of the state of Veracruz, Mexico were selected (19°15′ and 20°00′N, 96°15′ and 97°30′W) over a range of elevation covering 630 to 2000 m a.s.l. Of the sites selected to search for *P. townsendi*, 9 were historical and 13 were new sites, while for *T. pennatulus*, 7 were historical sites and 15 were new.

The sites were visited from one to three times between June and November 2010 which coincides with the rainy season in the region when it is easiest to detect the salamanders.

During each visit the microhabitats where *Parvimolge townsendi* and *Thorius pennatulus* are commonly found were carefully searched in two shifts (daytime: 1000 h–1600 h; nighttime 2000 h–0200 h). Sampling effort per visit was 16 person-hours (4 people×2 hours×2 shifts) and the cumulative sampling effort for the study was 672 person-hours (42 visits×16 person hours). Each salamander encountered was measured, weighed and photographed, and the microhabitat where it was found was noted.

To compare the observed abundance between sites, we calculated the rate of encounter (ER), weighting the number of salamanders found with the applied sampling effort (number of visits), as follows:
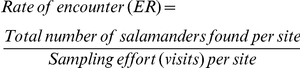
To determine if there was any dependence between the number of individuals found and the shift when they were found, or between the number of individuals and the microhabitat, we did a *X* ^2^ test [29] for *P. townsendi*. It was not possible to test for dependence for *T. pennatulus* owing to the paucity of data.

### Habitat description

At the sites where *Parvimolge townsendi* and *Thorius pennatulus* were found we measured air temperature during sampling. At each site we set up six 4×25 m plots (total area: 0.06 ha.) in which we surveyed the trees with a diameter at breast height (DBH)≥5 cm, measured their height and diameter, estimated the quantity of epiphytes on each tree and measured canopy cover. The latter was done by taking three digital photographs of the canopy (at one side, in the center and at the other side of each plot) from a height 1.3 m above the ground (Fujifilm FinePix S1800 camera), for a total of 18 photographs per site. The photos were processed following the method proposed by Korhonen *et al.* [30], using ImageJ software, version 1.43 [31] to calculate percentage canopy cover. Finally, in each plot, we measured the depth of the litter layer at 15 points (90 per site) and counted the number of tree trunks on the ground.

To detect any relationship between the habitat variables measured and the rate of encounter of each salamander, we used Spearman's rank correlation test [29] run in STATISTICA, version 7.0 [32].

### Potential distribution

To estimate the potential geographic distribution of each species we used MaxEnt, version 3.3.3a [27]; this time using both the historical and the new sites selected in this study, however only those that were geographically independent (i.e. separated by at least 1 km) were used. For the predictive model for *P. townsendi* we used 34 sites, while for that of *T. pennatulus* we used 15 sites. To generate the distribution models, we only used the environmental variables that were relevant according to MaxEnt, for which in a first analysis 19 environmental layers from WorldClim were used [28]. This way, it is possible to reduce overfitting the distribution models generated for each species [33]. Twelve environmental variables were used to generate the model for *P. townsendi* and ten were used for *T. pennatulus* (see Table S3). The logistic format was used to obtain the values of environmental suitability and the criterion of minimum training presence was used to convert the logistic values to binary presence-absence values. For *P. townsendi* the threshold value was 0.246, and for *T. pennatulus* it was 0.200. Additionally, using the ARCVIEW program, version 3.2 [34] we obtained the values for the environmental variables that together had a relative contribution greater than 80% to generate the model for each species. The values were obtained for all of the sites used in the models.

Once we had the potential distribution model for each species, we applied a filter in ARCVIEW 3.2 [34] to eliminate those areas predicted as having a suitable bioclimate for the salamanders, but that did not have the type of vegetation where they have been observed (i.e. cloud forest and tropical semideciduous forest, primary and secondary vegetation and shade coffee plantations [14], [15], [20]. The filter was defined based on the vegetation map for the state of Veracruz [35] and on the Series III map produced by INEGI [36] for the areas outside of the state of Veracruz.

## Results

### Salamanders collected in the past

For the 193 *P. townsendi* salamanders with information about collection year in the database used in this study, data observations were recorded in 22 different years between 1920 and 2008. The number of salamanders collected per year varied notably (range: 1–98 animals). Between 1969 and 1970 more than 70% (136 salamanders) of the total collected for this species was recorded, but for the majority of the years only one to four specimens were caught. For *T. pennatulus*, the 679 salamanders in the database with collection dates were recorded in 16 different years between 1869 and 2006. The number of salamanders caught varied markedly between years (1–72 specimens per year), and both 1940 and 1969 stand out with 302 and 233 *T. pennatulus* caught, respectively. Together, these two years represent nearly 80% of all of the individuals of this species recorded with a collection date.

### Current captures, habitat and microhabitat

**Figure 1:**
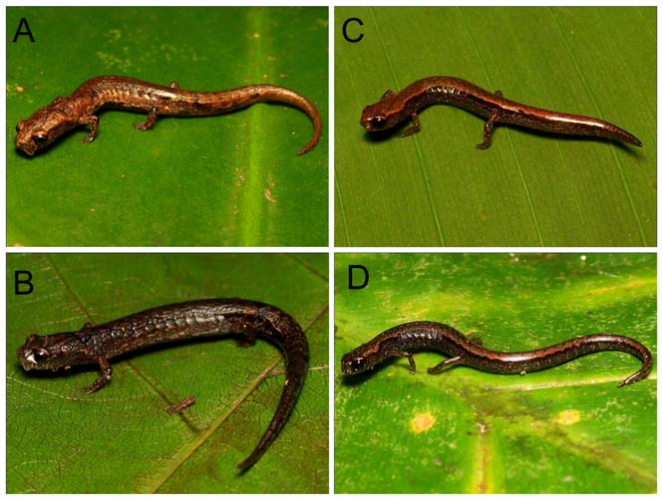
*Parvimolge townsendi* and *Thorius pennatulus*. A and B) Two *P. townsendi* males with different patterns of coloration (standard length = 20.7 and 23.8 mm, respectively). C and D) Two adult *T. pennatulus* salamanders (standard length = 17.8 and 16.8 mm, respectively).

A total of 201 *Parvimolge townsendi* and 13 *Thorius pennatulus* salamanders were recorded over the course of this study (Fig. 1), with a total sampling effort of 672 person hours. *P. townsendi* was recorded in 11 of the 22 sites visited, and seven of these 11 were historical (i.e. this species had been collected there in previous years) and the other four were sites where these salamanders were recorded for the first time. This species was not found in two of its historical localities, nor was a single *P. townsendi* seen in nine sites where there was no previous record of it, in spite of the search effort (Table S4). For *T. pennatulus* the 13 salamanders recorded were found in five of the 22 sites visited. Two of these five were historical, and the others were new sites. *T. pennatulus* was not found in five of the historical localities, nor was a single member of this species found in the 12 sites with no previous record (Table S4).

**Figure 2:**
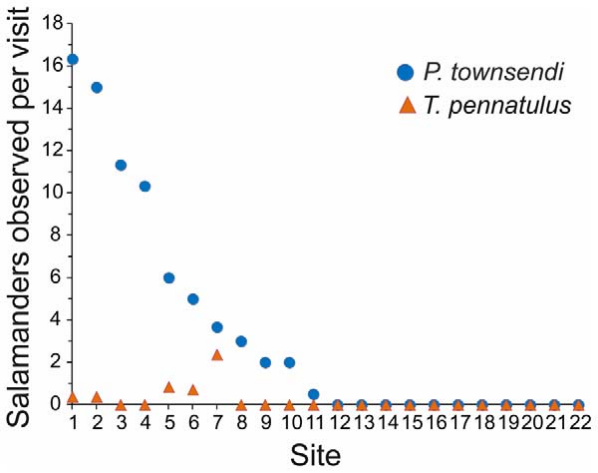
Rate of finding *Parvimolge townsendi* and *Thorius pennatulus* per site. Each visit represents a search effort of 16 person hours.

For *P. townsendi*, the mean rate of encounter in the eleven sites was 6.8 salamanders per visit (range: 0.5–16.3) (Fig. 2). The mean rate of encounter for *T. pennatulus* in five sites was 0.6 salamanders per visit (range: 0.3–2.3) (Fig. 2).

The sites where we encountered *P. townsendi* are forest fragments with a mean canopy cover of 84% to 88%, and located between elevations of 980 and 1950 m a.s.l. Nine of the sites were cloud forest fragments, some with shade coffee plantations inside the fragments, and the other two were tropical semideciduous forest with some coffee and banana plants (further details on habitat are given in Table S4). The sites where we found *T. pennatulus* were all cloud forest fragments with a mean canopy cover of 84% to 88%, located between 1290 and 1820 m a.s.l. (see Table S4 for more details about the habitat). In practically all survey sites, including those sites where we recorded both *P. townsendi* and *T. pennatulus*, there were signs of disturbance such as garbage, tree felling or agrochemical waste.

Of all the habitat characteristics evaluated, there was only a positive correlation between the rate of encounter for *P. townsendi* and litter depth (r_s_ = 0.632, p = 0.0368). For *T. pennatulus* none of the habitat variables evaluated were correlated with the rate of encounter.

**Figure 3:**
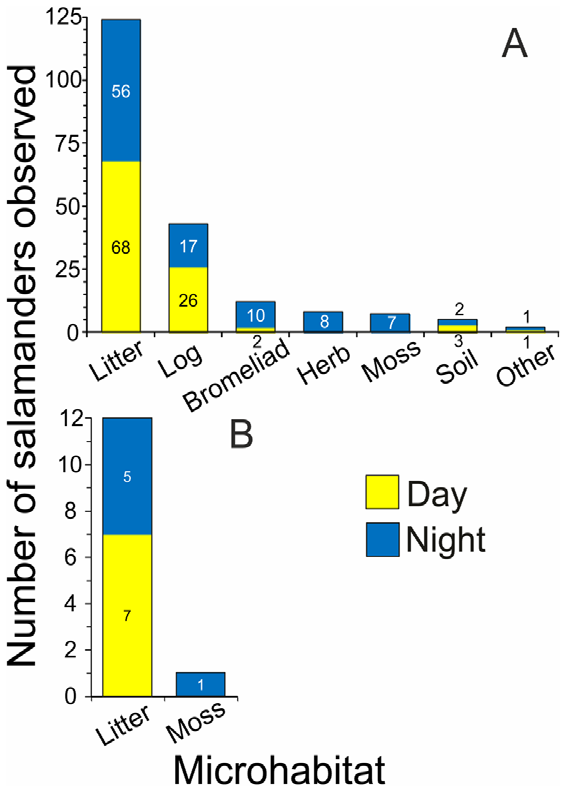
Microhabitat use. Number of *Parvimolge townsendi* (A) and *Thorius pennatulus* (B) salamanders found in different microhabitats and at different times of the day.

Half (50.5%) of the 201 *P. townsendi* salamanders found during this study were recorded during the daytime and the other half (49.5%) at night, so observed abundance for this species is independent of sampling time (χ^2^ = 0.0049, p = 0.943). *P. townsendi* was found in seven different microhabitats and observed abundance was found to be dependent on microhabitat (χ^2^ = 408.83, p<0.0001). Litter was by far the microhabitat where we found *P. townsendi* most often with 62% of all individuals observed, followed by fallen trunks, fallen bromeliads, herbaceous plants, moss, bare soil and others, respectively (Fig. 3). For *T. pennatulus*, seven of the 13 salamanders observed during this study were found during the day (and six at night), so for this species as well, observed abundance was independent of sampling time (χ^2^ = 0.076, p = 0.781). *T. pennatulus* was found in only two microhabitats, and exhibited a notable preference for litter over moss (Fig. 3). Observed abundance for this species was dependent on microhabitat (χ^2^ = 9.307, p = 0.002).

### Potential distribution

**Figure 4:**
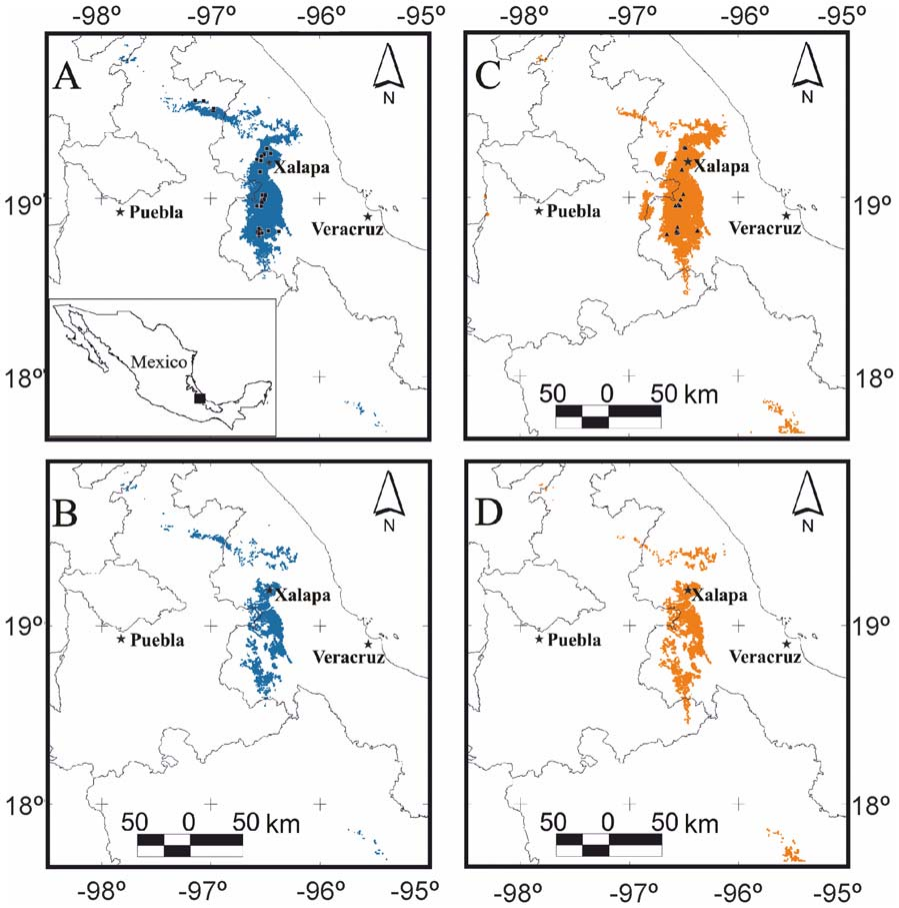
Potential distribution of *Parvimolge townsendi* and *Thorius pennatulus*. (A) Potential distribution area for *P. townsendi*; black square are the sites used in to generate the model, (B) Potential distribution are for *P. townsendi*, cropped to include only the appropriate vegetation (see Methods), (C) Potential distribution area for *T. pennatulus*; black triangles are the sites used in to generate the model and (D) Potential distribution area for *T. pennatulus*, cropped to include only the appropriate vegetation. The colored area is the predicted distribution area and the black stars indicate large cities.

According to the results of obtained using MaxEnt, the bioclimate is suitable for *P. townsendi* over an area of 3588 km^2^ that has an elongated shape running north-south, the majority of which falls within the state of Veracruz though it does include parts of the states of Puebla and probably Oaxaca and Hidalgo (Fig. 4). After eliminating the areas that have vegetation or types of land use where the species has not been recorded, the remaining area is 1605 km^2^; a 55% reduction in the area initially calculated. Of this 50% is explained by habitat loss and the remaining 5% corresponds to vegetation types where the species has not been recorded (Fig. 4). The potential distribution of *P. townsendi* is notably fragmented where cloud forest (including primary and secondary forest, the latter with shade coffee crops), accounts for approximately 77% of the predicted distribution area and tropical semideciduous forest (primary and secondary) accounts for 23%. For *T. pennatulus*, the predicted area that is bioclimatically suitable covers 4178 km^2^ and, as for *P. townsendi*, mostly falls within the state of Veracruz, with small areas in the states of Puebla, Oaxaca and Hidalgo (Fig. 4). On eliminating the areas with vegetation or types of land use where this species has not been recorded, the remaining area is 1713 km^2^; 59% less than the area initially calculated. In this case 48% can be attribute to habitat loss and the remaining 11% corresponds to vegetation types where the species has not been recorded (Fig. 4). The predicted distribution area is notably fragmented and is composed of 75% cloud forest (including primary and secondary forest) and 25% tropical semideciduous forest (primary and secondary).

There was overlap between the predicted areas of distribution for the two species: 87% of *P. townsendi*'s potential distribution coincides with that of *T. pennatulus*, while 75% of the area predicted for *T. pennatulus* coincides with that of *P. townsendi*.

**Figure 5:**
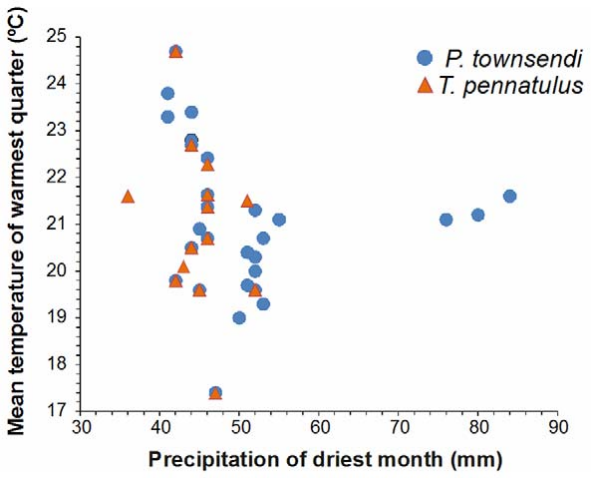
The environmental variables that make the greatest contribution in modeling the potential distribution of both species. Blue circles are the sites where the presence of *Parvimolge townsendi* was used for the model, and the orange triangles are those where the presence of *Thorius pennatulus* was used for the model.

The environmental variables that contributed more than 80% to the predictive model for *P. townsendi* were precipitation during the driest month, mean temperature of the warmest quarter, precipitation during the warmest quarter and annual variation in temperature (Table S3). For *T. pennatulus*, as for *P. townsendi*, precipitation during the driest month, mean temperature of the warmest quarter, and annual variation in temperature were the first, second and fourth most important bioclimate variables, but in this case third position was held by precipitation in the coldest quarter (Table S3). The relationship between the two most important bioclimate variables for generating the distribution models of both species are shown in Figure 5, where the high degree of similarity in the values of mean temperature of the warmest quarter can be observed and even have very similar ranges (17.4 to 24.7°C). For the precipitation of the driest month the values for *P. townsendi* cover a broader range, with a notable increase in the maximum (range: 41–84 mm) compared to the values for *T. pennatulus* (range: 36–52 mm).

## Discussion

In the present study we focused on two critically endangered salamander species, one of which was even thought to have gone extinct, by searching exhaustively in historical localities where they had been recorded, and also in new sites with habitats potentially suitable for them. We were able to confirm the presence of both species in the region, evaluate their abundance, estimate their distribution and also to detect populations not previously recorded. The approach we used to identify the current status of the two endangered salamander species may be also useful for studying other endangered species.

*Parvimolge townsendi* and *Thorius pennatulus* are in the same category of critically endangered (CR) according to the IUCN [1]; the highest risk category before extinction. However the results of this study suggest that these two species may be facing different levels of extinction risk. That of *P. townsendi* appears to be less worrisome than that of *T. pennatulus*, because the former was found in a greater number of sites, its total abundance was notably higher (as much as six times greater than that of *T. pennatulus*), it is capable of inhabiting environments with a certain degree of disturbance or management and exhibits greater plasticity in its use of microhabitats.

Sampling effort for the historical captures is not known for each species and the information given is only for the salamanders captured and does not include those seen. Thus, it is possible that *T. pennatulus* was abundant at some time in the past given that there were more historical captures of it than there were of *P. townsendi*. However in this study, with the same search effort, *T. pennatulus* was found to be very rare in practically all of the sites where it was observed. The low number of this species recorded during this study in comparison with the historical records lends support to the idea that it has undergone a drastic population decline in recent decades [3], [5], [14]. *P. townsendi*, which had been thought to be possibly extinct, was abundant or moderately abundant in the majority of the 11 sites where it was found, thus reversing this previous assessment. The populations of *P. townsendi* appear to be stable in the forest fragments where this species was found, although to confirm this it is necessary to carry out population studies over a longer period of time.

The differences in the abundance patterns detected for these two species in this study, as well as the number of localities where both species were found could be related, at least partially with the effects of the pathogenic chytrid fungus *Batrachochytrium dendrobatidis*. Cheng *et al.* [17] found that this fungus has been present in the region, specifically on Cerro Chicahuaxtla in central Veracruz since at least 1972. There, infected *T. pennatulus* have been found since 1972, while *P. townsendi* salamanders showed no evidence of this fungus in any of the specimens examined from the collections done in six different years between 1969 and 1983. These results could indicate that these two species differ in their degree of susceptibility to infection by this fungus which in turn has an effect on their survival rate. If this is the case then *P. townsendi* may be less vulnerable to infection by this chytrid and thus, its abundance would not be limited in the same way as that of *T. pennatulus*. This idea is supported by the data from Cerro Chicahuaxtla where historically dozens of specimens of both species were collected on several occasions in the mid 1970s and the late 1980s. After this, in spite of carrying out several searches in 1983, 1998 and 1999 [5] not a single salamander of either species was recorded. In our study, at the same locality and with a search effort of 48 person hours divided into three excursions during the rainy season we found nine *P. townsendi* salamanders, but not a single *T. pennatulus*. This might indicate the local extinction of *T. pennatulus*, and the reason for the apparent reappearance of *P. townsendi* might be related to our sampling design, or extreme population fluctuations in recent years that have led it to be very rare at certain times, as observed for other species [24].

The relationship between the different habitat characteristics and salamander abundance has been documented in several studies [37]–[40]. We found that the encounter rate for *P. townsendi* was only positively correlated with litter depth. Whitfield *et al.* [41] found a relationship between the decline in Neotropical terrestrial salamanders (and other herpetofauna) and a historical decrease in litter depth. Welsh & Droege [39] mention that the presence of litter is the most important condition for terrestrial salamanders since it is the microhabitat where they carry out the majority of their activities, including foraging, courting, and laying their eggs; all providing that humidity is relatively high. Although *P. townsendi* is not strictly terrestrial—given that it has been found in bromeliads and on herbaceous plants—this species tends to be found in litter and variations in this habitat could affect the number of animals found. While *T. pennatulus* can be considered an exclusively terrestrial species [13] there was no correlation between encounter rate and litter depth, which may be a result of the effect of the lower number of sites where this species was found (n = 5), rather than a biological effect.

Canopy cover is another attribute of the habitat that is generally positively correlated with salamander abundance because lower or no canopy cover reduces thermal buffering and increases evapotranspiration within the forest [39]. In a study done in the forests of North America by Welsh & Lind (cited in [39]) variation in canopy cover, which ranged from 62% to 83%, was closely related with plethodontid salamander abundance. In our study, no correlation was detected between the rate of encounter and canopy cover, but mean canopy cover varied only slightly (between 84% and 88%), and this might have been too narrow a range to detect any effect on the abundance of the salamanders we studied.

The potential distribution models generated in this study for both species indicate that their distribution is preferentially associated with cloud forest; there was more than 75% overlap in their potential distribution areas, and this coincides with values reported in the literature [12]–[15]. The predicted distributions for both species are restricted and fragmented, mainly in central Veracruz, though they do extend into some parts of the states of Puebla, Oaxaca and Hidalgo, where conditions similar to those required by these species occur.

The most significant environmental variables for generating the potential distribution models for both species support the idea that cold temperatures and high relative humidity are determinant for the majority of the plethodontids [39]. That said, *T. pennatulus* is found within a much narrower range of environmental conditions than *P. townsendi* is. If environmental variables that are important to the potential distribution of a species provide information about the environmental tolerance of that species [42], then it can be argued that *T. pennatulus* is more environmentally restricted, and is more sensitive to changes in its habitat.

The estimated decrease of more than 48% in the distribution area of both species as a result of habitat loss is worrisome, though it is a more conservative value than that given by Ochoa-Ochoa *et al.* [43]. Those authors calculated a loss of potential habitat for *P. townsendi* that was greater than 80% owing to changes in land use. The difference in these estimates might result from our including cloud forest and tropical semideciduous forest as appropriate types of vegetation for both species, along with secondary forest and shade coffee plantations (based on reports in several studies and our observations during this study), when we applied the vegetation filter to the first distribution model (see Methods). Given that habitat loss is very high, whether it is 48% or 80%, field work is necessary to detect the largest number of fragments that have populations of these species because they probably also house other microendemic species that might be facing challenges (see [44]). This could represent an opportunity for conservation: by protecting specific forest fragments it would be possible to preserve an assemblage of endangered species.

The potential distribution models we generated were useful not only for estimating the spatial distribution of the two species and the area of habitat lost as a result of conversion, they also served as a guide for detecting the sites that are environmentally suitable for these species, and where the field searches could be carried out. The models should be thought of as hypotheses on the distribution of these species and as such it is necessary to test them in the field to determine their degree of accuracy and reliability.

Finally, the methodological approach applied in this study and the results obtained highlight the importance of carrying out exhaustive searches in the field to get a better idea of the current situation of endangered species, particularly those that are critically endangered or even considered possibly extinct [22], [23]. It is also useful to identify those species that are genuinely in a critical situation from those for which there are no recent sampling data. This will allow us to gather the information required to better understand the problem of the dramatic decline around the world in amphibians.

## Supporting Information

Table S1 Data sources. List of the institutional collections with specimens of *Parvimolge townsendi* and *Thorius pennatulus* from which information was obtained for this study.
(DOC)

Table S2 Historic localities of *Parvimolge townsendi* and *Thorius pennatulus*. Geographic coordinates used in the first modeling of the potential distribution of each species in order to select the survey sites, and the number of salamanders that have been collected in each site in the past.
(DOC)

Table S3 Bioclimate layers used to generate the potential distribution models for each species. Percent contribution and permutation importance are given for each bioclimate variable.
(DOC)

Table S4 Characteristics of the sites surveyed during this study. Historic sites for *P. townsendi* (P), *T. pennatulus* (T) and for both species (B). CF is cloud forest, TSF is tropical semideciduous forest, and SC is shaded coffee plantations.
(DOC)
