# Supplementary material for: In Search of Critically Endangered Species: The Current Situation of Two Tiny Salamander Species in the Neotropical Mountains of Mexico
Source: PLoS One. 2012 Apr 2;7(4):e34023. doi: 10.1371/journal.pone.0034023 (PMC3317776; doi:10.1371/journal.pone.0034023)
Supplement: Table S1 — Data sources. List of the institutional collections with specimens of Parvimolge townsendi and Thorius pennatulus from which information was obtained for this study. (DOC) [file pone.0034023.s001.doc]

**Supporting Information**

**Table S1. Data sources**. List of the institutional collections with specimens of *Parvimolge townsendi* and *Thorius pennatulus* from which information was obtained for this study.

| **Institution** | **Collection code** | ***Parvimolge townsendi*** | ***Thorius pennatulus*** |
| --- | --- | --- | --- |
| Museum of Natural History (Division of Amphibians and Reptiles) | FMNH | 3 | 393 |
| Universidad Autónoma de México (Instituto de Biología) | CNAR | 4 | 6 |
| Universidad Autónoma de México (Museo de Zoología de la Facultad de Ciencias) | MZFC | 2 | - |
| Los Angeles County Museum of Natural History | Herps | 80 | 111 |
| Harvard University (Museum of Comparative Zoology) | MCZ-HU | 13 | 9 |
| University of Berkley (Museum of Vertebrate Zoology) | MVZ-herp | 74 | 153 |
| University of Colorado (Museum of Natural History) | Herps | 4 | - |
| University of Michigan - Ann Arbor (Museum of Zoology) | UMMZ | 10 | 1 |
| National Museum of Natural History | USMN | 19 | 130 |
| S/D | S/D | 10 | 12 |
| Academy of Natural Sciences | HRP | - | 15 |
| California Academy of Sciences (Herpetology Department) | Herp | - | 8 |
| Illinois Natural History Survey | Herps | - | 92 |
| Louisiana State University Museum of Natural Science | Herp | - | 1 |
| Texas Cooperative Wildlife Collections, Texas A & M University | TCWC | - | 2 |
| University of Kansas (Biodiversity Research Center) | KUH | - | 52 |
|  | **Total** | 219 | 985 |
