# Supplementary material for: In Search of Critically Endangered Species: The Current Situation of Two Tiny Salamander Species in the Neotropical Mountains of Mexico
Source: PLoS One. 2012 Apr 2;7(4):e34023. doi: 10.1371/journal.pone.0034023 (PMC3317776; doi:10.1371/journal.pone.0034023)
Supplement: Table S2 — Historic localities of Parvimolge townsendi and Thorius pennatulus. Geographic coordinates used in the first modeling of the potential distribution of each species in order to select the survey sites, and the number of salamanders that have been collected in each site in the past. (DOC) [file pone.0034023.s002.doc]

**Supporting Information**

**Table S2. Historic localities of *Parvimolge townsendi* and *Thorius pennatulus*.** Geographic coordinates used in the first modeling of the potential distribution of each species in order to select the survey sites, and the number of salamanders that have been collected in each site in the past.

|  |  |  |  | **Number of salamanders collected** | |
| --- | --- | --- | --- | --- | --- |
| **Locality** | **N. Latitude** | **W. Longitude** | ***Parvimolge townsendi*** | | ***Thorius pennatulus*** |
| **H1** | 18.8626 º | -97.0063 º | 69 | | 2 |
| **H2** | 18.8728 º | -97.0230 º | 29 | | 743 |
| H3 | 19.1427 º | -96.9869 º | 24 | | 12 |
| H4 | 18.8566 º | -97.0119 º | 24 | | - |
| H5 | 18.8583 º | -97.0166 º | 19 | | 36 |
| H6 | 18.8784 º | -96.8426 º | 10 | | 2 |
| H7 | 18.8861 º | -96.9993 º | 5 | | - |
| H8 | 18.8618 º | -96.9869 º | 5 | | - |
| H9 | 19.1500 º | -96.9666 º | 4 | | - |
| H10 | 19.1025 º | -97.0036 º | 3 | | 1 |
| H11 | 19.5426 º | -96.9137 º | 3 | | - |
| H12 | 19.0983 º | -97.0308 º | 2 | | - |
| H13 | 19.1844 º | -96.9572 º | 2 | | - |
| H14 | 19.1877 º | -96.9832 º | 2 | | - |
| H15 | 19.5333 º | -96.9666 º | 2 | | - |
| H16 | 19.4811 º | -96.9977 º | 2 | | - |
| H17 | 19.1264 º | -96.9858 º | 2 | | - |
| H18 | 19.9260 º | -97.3991 º | 2 | | - |
| H19 | 19.1416 º | -96.9927 º | 1 | | - |
| H20 | 18.9072 º | -97.0126 º | 1 | | 7 |
| H21 | 18.8425 º | -97.0131 º | 1 | | - |
| H22 | 18.8833 º | -96.9333 º | 1 | | - |
| H23 | 19.9042 º | -97.4036 º | 1 | | - |
| H24 | 19.9880 º | -97.4863 º | 1 | | - |
| H25 | 19.9927 º | -97.5547 º | 1 | | - |
| H26 | 19.1836 º | -96.96005 | 1 | | - |
| H27 | 19.3992 º | -96.9736 º | - | | 9 |
| H28 | 18.8488 º | -97.1049 º | - | | 8 |
| H29 | 19.5875 º | -96.9541 º | - | | 1 |
