# Supplementary material for: In Search of Critically Endangered Species: The Current Situation of Two Tiny Salamander Species in the Neotropical Mountains of Mexico
Source: PLoS One. 2012 Apr 2;7(4):e34023. doi: 10.1371/journal.pone.0034023 (PMC3317776; doi:10.1371/journal.pone.0034023)
Supplement: Table S3 — Bioclimate layers used to generate the potential distribution models for each species. Percent contribution and permutation importance are given for each bioclimate variable. (DOC) [file pone.0034023.s003.doc]

**Supporting Information**

**Table S3. Bioclimate layers used to generate the potential distribution models for each species.** Percent contribution and permutation importance are given for each bioclimate variable.

| **Bioclimate variable** | ***Parvimolge townsendi*** | | ***Thorius pennatulus*** | | |
| --- | --- | --- | --- | --- | --- |
|  | **Percent contribution (%)** | **Permutation importance (%)** | **Percent contribution (%)** | | **Permutation importance (%)** |
| 1. Precipitation of driest month | 37.3 | 34.1 | 37.6 | 37.3 | |
| 1. Mean temperature of warmest quarter | 32.6 | 4.2 | 31.4 | 8.1 | |
| 1. Precipitation of warmest quarter | 8.2 | 11 | 2.5 | 0 | |
| 1. Annual temperature range | 7 | 15.5 | 9.1 | 8.4 | |
| 1. Mean temperature of driest quarter | 4.9 | 0.3 | - | - | |
| 1. Precipitation of coldest quarter | 4.9 | 24 | 10 | 42 | |
| 1. Precipitation of wettest quarter | 2.9 | 7.6 | 2 | 3 | |
| 1. Temperature seasonality | 1.2 | 0.9 | 0.2 | 0.3 | |
| 1. Mean diurnal range | 0.6 | 0.8 | - | - | |
| 1. Mean temperature of wettest quarter | 0.1 | 0 | - | - | |
| 1. Minimum temperature of coldest month | 0.1 | 0.9 | - | - | |
| 1. Precipitation seasonality | 0.1 | 0.7 | 2.1 | 0 | |
| 1. Precipitation of wettest month | - | - | 2.7 | 0.2 | |
| 1. Annual precipitation | - | - | 2.3 | 0.6 | |
