# Supplementary material for: In Search of Critically Endangered Species: The Current Situation of Two Tiny Salamander Species in the Neotropical Mountains of Mexico
Source: PLoS One. 2012 Apr 2;7(4):e34023. doi: 10.1371/journal.pone.0034023 (PMC3317776; doi:10.1371/journal.pone.0034023)
Supplement: Table S4 — Characteristics of the sites surveyed during this study. Historic sites for P. townsendi (P), T. pennatulus (T) and for both species (B). CF is cloud forest, TSF is tropical semideciduous forest, and SC is shaded coffee plantations. (DOC) [file pone.0034023.s004.doc]

**Supporting Information**

**Table S4. Characteristics of the sites surveyed during this study.** Historic sites for *P. townsendi* (P), *T. pennatulus* (T) and for both species (B). CF is cloud forest, TSF is tropical semideciduous forest, and SC is shaded coffee plantations.

| **Site** | **N. Lat.** | **W. Long.** | **Type of vegetation** | **Number of visits** | **Sampling efforts (hours)** | **Number of salamanders observed** | |
| --- | --- | --- | --- | --- | --- | --- | --- |
|  |  |  |  |  |  | ***Parvimolge townsendi*** | ***Thorius pennatulus*** |
| 1^P^ | 19.187º | -96.962º | CF-SC | 3 | 48 | 49 | 1 |
| 2^B^ | 19.091º | -96.995º | CF | 3 | 48 | 45 | 1 |
| 3 | 19.383º | -97.003º | CF | 1.5 | 24 | 17 | 0 |
| 4 | 19.517º | -96.994º | CF | 3 | 48 | 31 | 0 |
| 5 | 19.490º | -97.033º | CF | 2.5 | 40 | 15 | 2 |
| 6^T^ | 19.582º | -96.944º | CF | 3 | 48 | 15 | 2 |
| 7^P^ | 19.092º | -97.031º | CF | 3 | 48 | 11 | 7 |
| 8^B^ | 18.858º | -97.011º | TSF | 3 | 48 | 9 | 0 |
| 9^B^ | 19.108º | -96.994º | CF | 3 | 48 | 6 | 0 |
| 10^P^ | 19.127º | -96.987º | CF | 1 | 16 | 2 | 0 |
| 11^B^ | 18.901º | -97.012º | TSF-SC | 2 | 32 | 1 | 0 |
| 12^B^ | 18.871º | -97.028º | TSF | 2 | 32 | 0 | 0 |
| 13 | 19.134º | -97.013º | CF | 1 | 16 | 0 | 0 |
| 14 | 18.897º | -96.861º | TSF | 2 | 32 | 0 | 0 |
| 15 | 19.019º | -96.920º | CF | 1 | 16 | 0 | 0 |
| 16 | 19.679º | -96.853º | CF | 1 | 16 | 0 | 0 |
| 17 | 19.682º | -96.828º | CF | 2 | 32 | 0 | 0 |
| 18^B^ | 18.860º | -96.993º | TSF | 1 | 16 | 0 | 0 |
| 19 | 18.812º | -97.058º | CF | 1 | 16 | 0 | 0 |
| 20 | 19.101º | -97.112º | CF | 1 | 16 | 0 | 0 |
| 21 | 19.797º | -97.215º | CF | 1 | 16 | 0 | 0 |
| 22 | 19.836º | -97.221º | CF | 1 | 16 | 0 | 0 |
